# Seascape Connectivity Shapes Genetic and Species β‐Diversity in Tropical Reef Fishes

**DOI:** 10.1002/ece3.73760

**Published:** 2026-06-04

**Authors:** Maurine Vilcot, Thomas Keggin, Camille Albouy, Giulia F. A. Donati, Andrés Acosta‐Chaparro, Giomar H. Borrero‐Pérez, Marie‐Charlotte Cheutin, Juan David González Corredor, Régis Hocdé, Jean‐Baptiste Juhel, Virginie Marques, David Mouillot, Maria Mutis‐Martinezguerra, Andrea Polanco F., Mark J. A. Vermeij, Loïc Pellissier, Stéphanie Manel

**Affiliations:** ^1^ CEFE, Univ Montpellier, CNRS, EPHE‐ PSL University Montpellier France; ^2^ Department of Environmental Systems Science, Ecosystems and Landscape Evolution, Institute of Terrestrial Ecosystems ETH Zürich Zürich Switzerland; ^3^ Swiss Federal Institute for Forest, Snow and Landscape Research WSL Birmensdorf Switzerland; ^4^ EAWAG Swiss Federal Institute of Aquatic Science & Technology Dübendorf Switzerland; ^5^ Programa de Biodiversidad y Ecosistemas Marinos, Instituto de Investigaciones Marinas y Costeras‐INVEMAR, Museo de Historia Natural Marina de Colombia (MHNMC) Santa Marta Colombia; ^6^ Independent Researcher Santa Marta Colombia; ^7^ MARBEC Univ Montpellier, CNRS, Ifremer, IRD Montpellier France; ^8^ RSMAS, University of Miami Key Biscayne Florida USA; ^9^ LEEISA, CNRS, IFREMER, Université de Guyane Cayenne France; ^10^ Laboratorio de Ecología Litoral, Instituto de Ciencias Marinas y Limnológicas Facultad de Ciencias, Universidad Austral de Chile Valdivia Chile; ^11^ Fundación Biodiversa Colombia Bogotá Colombia; ^12^ Department of Freshwater and Marine Ecology, Institute for Biodiversity and Ecosystem Dynamics University of Amsterdam Amsterdam the Netherlands; ^13^ Carmabi Foundation Willemstad Curaçao; ^14^ Institut Universitaire de France Paris France

**Keywords:** connectivity, isolation by distance, marine fish, species‐genetic diversity correlation, β‐diversity

## Abstract

Habitat configuration governs the movement of organisms across landscapes, thereby shaping both population structure and community assembly. While theoretical and empirical studies have assessed how habitat connectivity simultaneously influences intra‐ and interspecific diversity, direct comparisons across contrasting biogeographic regions remain limited. Here, we investigate patterns of genetic and species β‐diversity in tropical reef fishes across two ocean basins with distinct spatial configurations: the Caribbean Sea and the Western Indian Ocean. Using a comparative framework based on species occurrence data from five fish families and single nucleotide polymorphism (SNP) data from 19 species, we detected significant isolation by distance at both population and community levels in the Western Indian Ocean, but only at the community level in the Caribbean Sea. Additionally, genetic and species β‐diversity were positively correlated among species in the Western Indian Ocean, but not in the Caribbean Sea. Together, these results suggest that the shorter inter‐reef distances of the Caribbean Sea promote higher connectivity, leading to a decoupling of intra‐ and interspecific β‐diversity patterns.

## Graphical abstract

Our study combines species occurrence data from five reef fish families with genomic SNP data for 19 species. A correlation between genetic and species β‐diversity emerges only in the Western Indian Ocean, where higher genetic β‐diversity and significant isolation by distance at both the population and community levels are detected. In contrast, the stronger reef connectivity among Caribbean reefs leads to a decoupling between intra‐ and interspecific dissimilarity patterns.

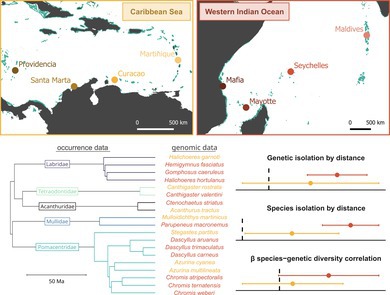

## 1 Introduction

Dispersal is a key evolutionary process that influences the spatial structure of populations and communities (Kokko and López‐Sepulcre 2006). Through the exchange of individuals among patches of habitat, dispersal determines gene flow (Hellberg 2009) and species colonization dynamics (Cowman and Bellwood 2013), shaping both genetic composition between populations and species composition between communities. Hence, when neutral processes such as dispersal serve as important drivers of the distribution of biodiversity across scales of organization, a covariation between genetic and species compositional dissimilarities is expected. Species‐genetic diversity correlations (SGDCs) quantify such patterns (Vellend 2005), and may reveal common ecological and evolutionary processes that shape biodiversity from populations to communities (Lamy et al. 2017). However, biodiversity patterns are influenced by intrinsic biological traits (e.g., Donati et al. 2021; Keggin et al. 2023) and extrinsic properties (e.g., Liggins et al. 2016) that can modulate the association between intra‐ and interspecific diversity (Vilcot et al. 2024).

Landscape connectivity, shaped by the isolation among suitable habitats, can impose significant constraints on functional connectivity between communities, with patches that are isolated by distance or by dispersal barriers requiring greater dispersal capacities for successful colonization (Suárez et al. 2022). Limited connectivity often increases dissimilarity with geographic distance. This pattern, known as “isolation by distance” (IBD) in population genetics (Kimura and Weiss 1964; Wright 1943) or “distance decay” in community ecology (Whittaker 1960, 1972; Nekola and White 1999), depends on the effective number of migrants, as well as on population or community size. IBD is used as a null model to test the effect of distance and dispersal on intra‐ and interspecific compositional dissimilarity (i.e., β‐diversity). Early work on SGDCs extended this framework to β‐diversity, showing a parallel decrease in community similarity with geographic distance at both genetic and species levels (Baselga et al. 2013). Such similar patterns are expected when diversity distribution is constrained by the neutral process of dispersal (Baselga et al. 2015), yet empirical evidence remains scarce, especially in marine systems. Given that the slope of IBD is often considered a good indicator of functional connectivity (Selkoe and Toonen 2011; Benestan et al. 2021), comparing these slopes across contrasting environments can help understand the role of geographic factors on dissimilarity patterns across scales of biological organization.

In a marine environment, the exchange of genes and species partly depends on seascape connectivity (Galindo et al. 2006; Hansen and Hemmer‐Hansen 2007; Manel and Holderegger 2013; Selkoe et al. 2016). The structural component of this connectivity depends on the spatial configuration of reefs and open ocean and the hydrological connectivity driven by ocean currents (Olds et al. 2016). Reefs act as stepping stones that favor connectivity (Kimura and Weiss 1964; Buonomo et al. 2017; Boulanger et al. 2020), whereas water trenches can hinder movement and gene flow among populations (Huyghe and Kochzius 2018). Currents can further facilitate or restrict the movement of larvae and juveniles across the seascape, thereby linking distant communities or separating close habitat patches (D'Aloia et al. 2015). Together, these factors (geographic distance, currents, and habitat structure) create a complex mosaic of connectivity that shapes the population dynamics and evolutionary trajectories of marine organisms.

Marine bioregions contain distinct species assemblages (Spalding et al. 2007), but share lineages from recent global radiations (Rabosky et al. 2018) and common biological traits (McLean et al. 2021). Exploring diversity patterns across bioregions provides a natural experiment with comparable communities containing phylogenetically related species that share similar life‐history traits, while evolving within different seascape configurations (Kulbicki et al. 2013; Parravicini et al. 2021). In our study, we examine and compare genetic and species β‐diversity patterns between the Caribbean Sea and the Western Indian Ocean. These two marine basins represent unique biogeographic environments with varying species‐specific connectivity levels that can influence spatial differences in both community structure and intraspecific genetic similarity. The Caribbean Sea has a higher proportion of suitable habitats for reef fish, with a smaller mean distance among habitat patches (Keggin 2023), which reflects higher potential connectivity than the Western Indian Ocean.

Here, we aim to investigate how habitat connectivity shapes both population and community structure by integrating fish datasets from the Caribbean Sea and the Western Indian Ocean. We expect β‐diversity patterns to exhibit IBD, leading to a positive correlation between species and genetic β‐diversity in regions with more limited dispersal, specifically in the Western Indian Ocean but not in the Caribbean Sea.

## 2 Material and Methods

### 2.1 Sampling Design

**FIGURE 1:**
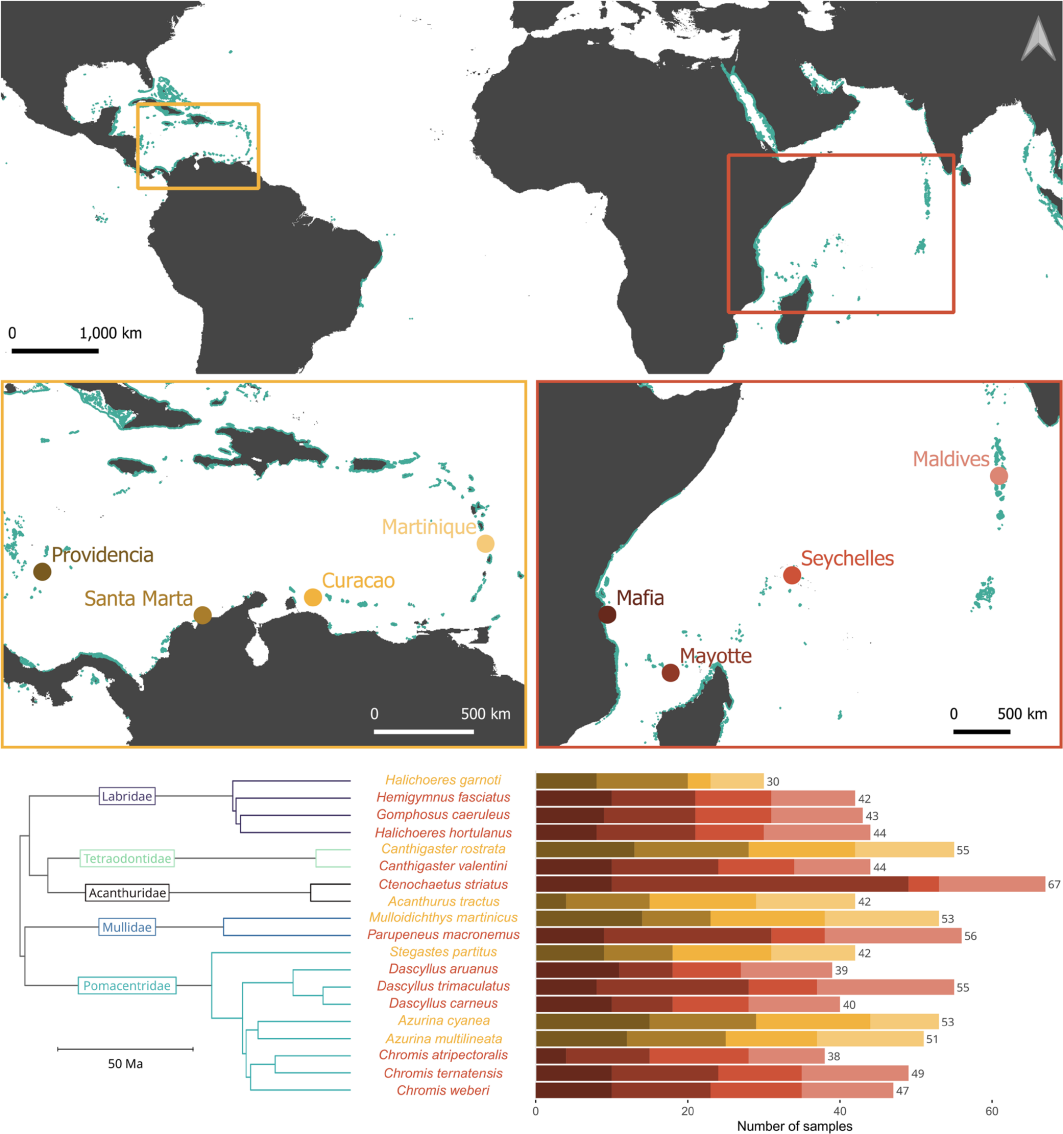
Sampling scheme of the 19 genotyped species. Sampling sites in the Caribbean Sea (in orange, from darkest to lightest: Providencia, Santa Marta, Curaçao, Martinique) and the Western Indian Ocean (in red, from darkest to lightest: Mafia, Mayotte, Seychelles, Maldives), with reefs shown in shades of blue (UNEP‐WCMC et al. 2021). Phylogenetic relationships spanning five families (Rabosky et al. 2018; Chang et al. 2019): Acanthuridae (black), Labridae (purple), Mullidae (blue), Pomacentridae (turquoise), Tetraodontidae (light blue). Number of samples after genetic filtering, for each species at each sampling site. Adapted from Donati et al. (2021) and Keggin et al. (2023).

To assess the influence of varying seascape configurations and connectivity levels on biodiversity, we compared patterns of genetic and species fish diversity across two contrasting ocean basins: the Western Indian Ocean and the Caribbean Sea. In the Western Indian Ocean, we obtained genetic data from Donati et al. (2021) which included samples from four sites: the Republic of Maldives, Mafia Island (Tanzania), the Seychelles, and Mayotte Island (the Comoros archipelago) (range of distances between sites: 805–3931 km). In the Caribbean Sea, we obtained genetic data from Keggin et al. (2023) which also contain samples from four sites: Providencia Island (Colombia), Santa Marta (Colombia), Curaçao, and Martinique (France) (range of distances between sites: 556–2190 km; Figure 1).

These ocean basins were selected due to their contrasting seascape configurations. According to the Marine Ecoregions of the World (MEOW) defined by Spalding et al. (2007), the four Caribbean Sea sites are located within a single province (Tropical Northwestern Atlantic), while the four Western Indian Ocean sites are located in two distinct provinces (Central Indian Ocean Islands and Western Indian Ocean). As described in Keggin et al. (2023), which quantified seascape metrics at the provinces scale, the Caribbean Sea exhibits a greater habitable reef area than the Western Indian Ocean (383,013 vs. 248,661 km^2^) over a smaller total area (6,899,039 vs. 14,822,619 km^2^). Additionally, the Caribbean Sea contains a higher number of habitat patches, which are on average larger (2350 vs. 1884 km^2^; Keggin 2023). These structural differences result in a smaller mean distance among habitat patches in the Caribbean Sea compared to the Western Indian Ocean (23 vs. 39 km).

### 2.2 Genetic Data

For the species‐level diversity, we targeted 27 species in the Western Indian Ocean and 15 species in the Caribbean Sea, which were sampled in 2016–2017 and in 2018–2020, respectively (Donati et al. 2021; Keggin 2023). To ensure taxonomic comparability, we prioritized families that have species in both ocean basins. The total of 42 reef fish species included in the genetic dataset was also selected to ensure maximum representation across a broad range of ecological and morphological traits, including body weight (ranging from 0.22 g for *Dascyllus aruanus* to 6315 g for *Caranx melampygus* across sampled individuals), morphology (see Donati et al. 2025 for species of the Western Indian Ocean), and distribution within the Teleost phylogeny. Fish sampling was conducted via scuba diving using hand barrier nets. For larger‐bodied and more mobile species, we complemented the sampling with locally caught fish from local markets. For each specimen, we collected tissue samples from either the muscles or the fins and stored them in either 90% EtOH or RNAlater. In total, across the 42 species, we sampled 1701 individuals, with 1061 from the Western Indian Ocean and 640 from the Caribbean Sea (Figure S1).

Full details of genetic data and metric preparation can be found in Donati et al. (2021) and Keggin et al. (2023). In summary, DNA extraction from tissue samples was carried out using the LGC sbeadex tissue purification kit and the KingFisher Flex by Thermo Fisher Scientific for large extraction batches. Libraries for double digest restriction‐site‐associated sequencing (ddRADseq) were constructed following a similar protocol adapted from Peterson et al. (2012) as described in Donati et al. (2021) for the Western Indian Ocean, and in Keggin et al. (2023) for the Caribbean Sea. Briefly, libraries were prepared using EcoRI and TaqI restriction enzymes. A set of 48 barcodes, combined with two Illumina indexes, was used to tag samples, generating 12 uniquely indexed equimolar pools for the Western Indian Ocean and 19 for the Caribbean Sea. Negative controls and individual replicates were included to detect contamination and assess sequencing consistency. All Caribbean Sea libraries were pooled together while maintaining equimolarity across samples. Western Indian Ocean libraries were sequenced on 12 lanes on the Illumina HiSeq 2500 (2 × 125 bp), while Caribbean Sea libraries were sequenced on one lane of the Illumina NovaSeq 6000 (2 × 150 bp).

For each species separately, SNP calling was carried out according to Donati et al. (2021), but we used the post‐genotyping filtering option from Keggin et al. (2023). Specifically, we excluded individuals with more than 50% of missing data, and SNPs with more than 20% of missing data in each sampling site or 5% of missing data across all individuals. Additionally, only the first SNP per locus was retained to reduce bias across loci. Given the different genomic architectures of each species, differences in SNP numbers were expected across species. To control for this bias, we randomly subsampled loci for each species to the minimum number of loci found across species in both oceans (*n* = 2852). To address the unequal numbers of genotyped individuals between species and sampling sites, we also subsampled each site to a maximum of 10 individuals per sampling site per species. The SNP and individual subsampling steps, and all derived metric calculations, were carried out iteratively 999 times. After confirming an acceptably low level of variation between iterations, the mean metric values across these 999 iterations were used in the final analyses.

Among the 42 species genotyped, we retained only those for which genetic data were available for at least three individuals in each of the four sampling sites, and samples with a minimum depth of coverage of 4. We further only retained species which had family representatives left in both oceans. Over the 42 species, 10 were excluded because they lacked genetic data in at least one site across the four sampled sites, and an additional 13 species were excluded because they had no family relatives remaining in both oceans (Table S1). This resulted in a reduced final selection of 890 individuals from 19 species spanning five families: the Acanthuridae (surgeonfishes, tangs, unicornfishes), Labridae (wrasses), Mullidae (goatfishes), Pomacentridae (damselfishes), and Tetraodontidae (puffers; Figure 1).

### 2.3 Occurrence Data

For the community‐level diversity, we obtained occurrence data for 15,595 Actinopterygii and Chondrichthyes species through an updated version of Albouy et al. (2019) dataset. This dataset combines the GASPAR database (Parravicini et al. 2013) and the Ocean Biogeographic Information System (OBIS, http://www.obis.org) utilizing a 1° resolution grid covering all oceans. To parallel the genetic sampling, we extracted the list of fish species present at each sampling site within a 111 km buffer (~1° at the equator) from this global database. The extraction identified a total of 2239 species in the four Caribbean Sea sites and 2518 species in the Western Indian Ocean sites. From this list of species presence at each site, we generated lists of species per site for each selected family.

### 2.4 Genetic and Species β‐Diversity

Genetic β‐diversity (β‐GD) for each genotyped species was computed as Hedrick's *G*″_ST_ (Meirmans and Hedrick 2011) as described in Keggin et al. (2023). The original *G*_ST_, defined as $\frac{H_{T}-H_{S}}{H_{T}}$ (Nei 1987), measures genetic differentiation among populations and is similar to the common *F*_ST_, though it accommodates multiple alleles. Hedrick's *G*'_ST_ = $\frac{G_{\mathrm{ST}}}{G_{\mathrm{ST}\left(\max\right)}}$ (Hedrick 2005) standardizes *G*_ST_ by the maximum possible value it can obtain. The unbiased estimator *G*″_ST_ includes an additional correction to reduce bias that can occur when the number of sampled populations is small (Meirmans and Hedrick 2011). We computed both overall and pairwise values between sampling sites by using the *Gst_Hedrick* and the *pairwise_Gst_Hedrick* functions of the “mmod” R package (Winter 2012). The reported β‐GDs are the average of all the 999 resampling of the 2852 SNPs datasets.

We quantified species β‐diversity (β‐SD) for each selected family using Jaccard's dissimilarity index (β_jac_‐SD), which measures how different two sites are in terms of species composition. We opted for Jaccard's dissimilarity index as it gives more weight to unique species in each site than Sørensen dissimilarity index for example (Baselga 2012). We further partitioned the β_jac_‐SD into its nestedness component (β_jne_‐SD), reflecting differences due to species richness, and its turnover (β_jtu_‐SD) component, which captures species replacement and is independent of richness differences (Baselga 2010). These calculations were performed with the “betapart” R package (Baselga and Orme 2012), using the function *beta.multi* for overall estimates and the function *beta.pair* for pairwise estimates between sites.

Variation in dispersal potential for each family in each ocean basin was estimated with the pelagic larval duration (PLD). PLD values for each species present in our sampling sites were taken from Luiz et al. (2013). If not available at the species level, the PLD was computed as the median at the genus level.

### 2.5 Genetic and Species Isolation by Distance

We assessed population and community IBD, that is the increase of genetic and species β‐diversity with geographic distance (Wright 1943; Kimura and Weiss 1964; Nekola and White 1999), within each ocean basin. To integrate our multi‐taxa dataset, we implemented maximum‐likelihood population‐effects (MLPE) linear mixed models (Clarke et al. 2002). MLPE includes a covariate structure to account for the nonindependence of pairwise values (β‐diversity or geographic distance), and offers greater flexibility than traditional Mantel tests as it allows to add a random effect for species or families. This enabled us to estimate an average IBD slope for each ocean, while permitting each species to have its own baseline level of β‐diversity (i.e., different intercepts). We estimated the geographic distance (in km) as the shortest in‐water distance between pairs of sites avoiding land masses, using the GEBCO‐gridded bathymetry data (https://www.gebco.net) and the *lc.dist* function of the “marmap” R package (Pante and Simon‐Bouhet 2013). We fitted the MLPE models using the *lme* function from the “nlme” R package combined with the *corMLPE* function of the “corMLPE” R package. The marginal (*R* ^2^m) and conditional (*R* ^2^c) coefficients of determination were computed using the *r.squaredGLMM* function of the “MuMIn” R package.

### 2.6 β Species‐Genetic Diversity Correlations

To investigate the relationship between genetic and species dissimilarity, we aimed to quantify β species‐genetic diversity correlations (β‐SGDCs) for each genotyped species. Similarly to the IBD, we tested β‐SGDC within each ocean using MLPE linear mixed models, combining distance matrices of all species/family from each ocean basin, with a random effect on the species/family.

All statistical analyses were performed in R v4.1.2 (R Core Team 2022).

## 3 Results

### 3.1 Genetic and Species Diversities

**FIGURE 2:**
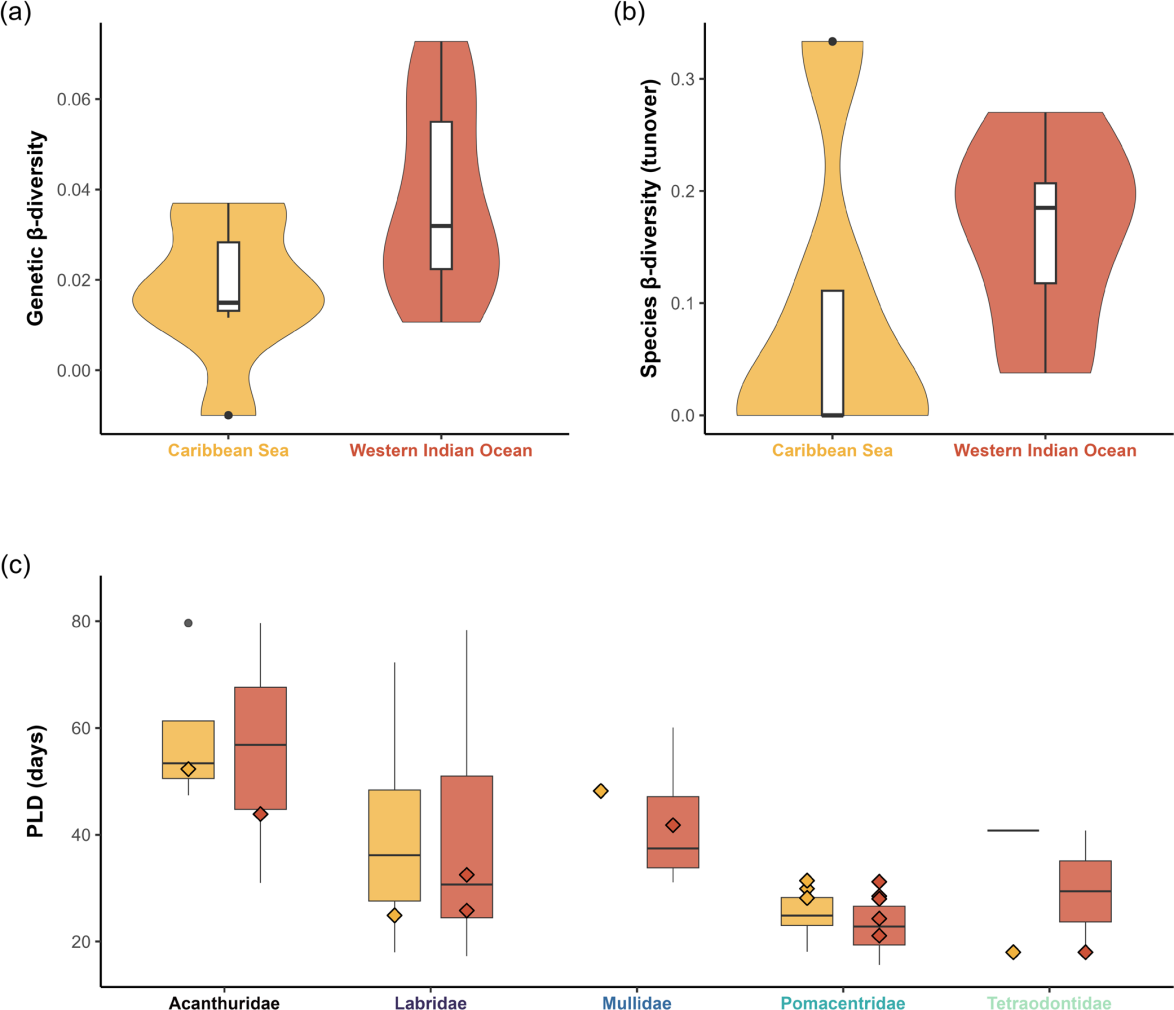
Comparison of genetic β‐diversity, species β‐diversity, and trait diversity between the Caribbean Sea (orange) and Western Indian Ocean (red). (a) Genetic β‐diversity was measured as genetic differentiation (*G*″_ST_) for each species and is adapted from Keggin et al. (2023). (b) Species β‐diversity was measured as Jaccard turnover (β_jtu_‐SD) for each family. (c) Trait diversity was measured as pelagic larval duration (PLD). Diamond shapes are PLD for species of the genetic dataset, and boxplots are the PLD for species of the community dataset for which information was available from Luiz et al. (2013).

Across the 19 genotyped species (Table 1), genetic β‐diversity was on average lower (Figure 2a; ANOVA *F* = 4.77, *p*‐value = 0.043) in the Caribbean Sea (mean β‐GD = 0.0178 ± 0.016) than in the Western Indian Ocean (mean β‐GD = 0.0379 ± 0.021). With only five families compared at the community level, we found no significant difference of β_jtu_‐SD (Figure 2b; ANOVA *F* = 0.96, *p*‐value = 0.355) between the Caribbean Sea (mean β_jtu_‐SD = 0.089 ± 0.145) and the Western Indian Ocean (mean β_jtu_‐SD = 0.163 ± 0.089). Three families in the Caribbean Sea (Acanthuridae, Mullidae and Labridae) exhibited no species turnover between most pairs of sites (i.e., β_jtu_‐SD = 0 for all site pairs), such that the total Jaccard dissimilarity (β_jac_‐SD) was entirely explained by nestedness for these families (Table 1).

**TABLE 1:** Within‐family β species diversity (β_jac_‐SD) and within‐species β genetic diversity (β‐GD) metrics in each ocean (values from Keggin 2023), with indication of the number of species in the presence data (*N* species), and the number of individuals in the genetic data (*N* individuals), for each ocean: Caribbean Sea and the Western Indian Ocean.

Family	Ocean	*N* species	β_jac_‐SD	β_jtu_‐SD	β_jne_‐SD	Species	*N* individuals	β‐GD
Acanthuridae	Caribbean Sea	5	0.400	0.000	0.400	*Acanthurus tractus*	42	0.0350
	Western Indian Ocean	42	0.410	0.207	0.203	*Ctenochaetus striatus*	69	0.0106
Labridae	Caribbean Sea	32	0.406	0.000	0.406	*Halichoeres garnoti*	28	0.0149
	Western Indian Ocean	93	0.437	0.270	0.167	*Gomphosus caeruleus*	44	0.0172
						*Halichoeres hortulanus*	46	0.0202
						*Hemigymnus fasciatus*	44	0.0727
Mullidae	Caribbean Sea	5	0.200	0.000	0.200	*Mulloidichthys martinicus*	53	−0.0100
	Western Indian Ocean	17	0.286	0.118	0.168	*Parupeneus macronemus*	56	0.0354
Pomacentridae	Caribbean Sea	21	0.284	0.111	0.172	*Azurina cyanea*	54	0.0147
						*Azurina multilineata*	51	0.0370
						*Stegastes partitus*	46	0.0216
	Western Indian Ocean	71	0.487	0.185	0.302	*Chromis atripectoralis*	35	0.0654
						*Chromis ternatensis*	49	0.0435
						*Chromis weberi*	45	0.0631
						*Dascyllus aruanus*	39	0.0522
						*Dascyllus carneus*	55	0.0232
						*Dascyllus trimaculatus*	40	0.0231
Tetraodontidae	Caribbean Sea	12	0.429	0.333	0.095	*Canthigaster rostrata*	57	0.0116
	Western Indian Ocean	22	0.363	0.038	0.325	*Canthigaster valentini*	44	0.0284

To account for differences in dispersal abilities of focal families that could underlie differences in β‐diversity between oceans, we verified that the average PLD across species retained for biodiversity assessments did not differ between ocean basins. There was no difference in the average PLD between oceans for species included in the community dataset (Figure 2c; *F* = 0.17, *p*‐value = 0.678), nor for those composing the genetic dataset (Figure 2c; *F* = 0.57, *p*‐value = 0.464).

### 3.2 Genetic Isolation by Distance

**FIGURE 3:**
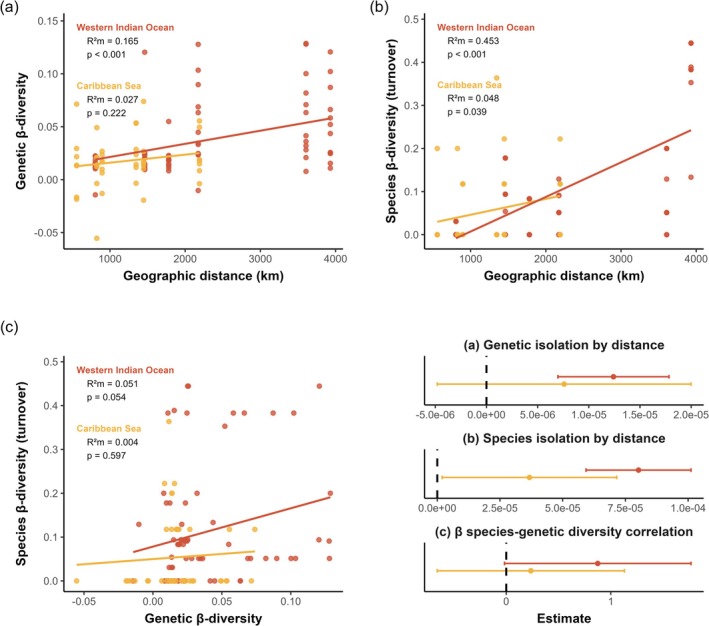
Contrasting isolation by distance and species‐genetic diversity correlation between the Caribbean Sea (orange) and the Western Indian Ocean (red) using MLPE linear mixed model. Isolation by distance on species β‐diversity (a) and on genetic β‐diversity (b). (c) β species‐genetic diversity correlation between species β‐diversity and genetic β‐diversity. Species β‐diversity was measured as Jaccard turnover (β_jtu_‐SD) for each family and genetic β‐diversity was measured as genetic differentiation (*G*"_ST_) for each species.

We evaluated the impact of geographic distance on genetic β‐diversity in each ocean basin separately (Figure 3a). For within‐species β‐GD, we found evidence of IBD across species of the Western Indian Ocean, where genetic dissimilarity increased significantly with geographic distance (Table 2; MLPE *p*‐value < 0.001). This pattern suggests restricted gene flow among populations as geographic distance increases. In contrast, we detected no significant IBD across species of the Caribbean Sea (Table 2; MLPE *p*‐value > 0.05), indicating that gene flow may be higher or not dependent on geographic distance in this region.

**TABLE 2:** Summary statistics of the MLPE linear mixed models by ocean basin for isolation by distance (IBD) and β species‐genetic diversity correlation (β‐SGDC). MLPE are between genetic β‐diversity (β‐GD), species turnover (β_jtu_‐SD), species total dissimilarity (β_jac_‐SD), and geographic distance (in km). *R* ^2^c: Conditional *R* ^2^; *R* ^2^m: Marginal *R* ^2^. Significant relations (*p*‐value < 0.05) are indicated in bold.

Hypothesis	Response	Predictor	Ocean	Estimate	*p*	*R* ^2^_m_	*R* ^2^_c_
Genetic IBD	β‐GD~	Geographic distance	Caribbean Sea	7.59E‐06	0.222	0.03	0.29
			**Western Indian Ocean**	**1.24E‐05**	**0.000**	0.17	0.25
Species IBD	β_jtu_‐SD~	Geographic distance	**Caribbean Sea**	**3.68E‐05**	**0.039**	0.05	0.52
			**Western Indian Ocean**	**8.02E‐05**	**0.000**	0.45	0.45
	β_jac_‐SD~	Geographic distance	**Caribbean Sea**	**9.17E‐05**	**0.000**	0.11	0.11
			**Western Indian Ocean**	**6.90E‐05**	**0.000**	0.47	0.67
β‐SGDC	β_jtu_‐SD~	β‐GD	Caribbean Sea	0.235	0.597	0.00	0.47
			Western Indian Ocean	0.875	0.054	0.05	0.05
	β_jac_‐SD~	β‐GD	Caribbean Sea	0.836	0.093	0.02	0.02
			**Western Indian Ocean**	**0.852**	**0.010**	0.07	0.07

### 3.3 Species Isolation by Distance

We also tested for a signal of IBD on within‐family species β‐diversity for each ocean (Figure 3b). Using the species turnover β_jtu_‐SD, we detected a significant relationship between within‐family β_jtu_‐SD and geographic distance both in the Western Indian Ocean and in the Caribbean Sea (Table 2). Results using total species dissimilarity (β_jac_‐SD) were similar (Figure S2). This suggests that species total dissimilarity and turnover within families are spatially structured by distance in both oceans.

### 3.4 Species‐Genetic Diversity Correlation

To test whether genetic and species β‐diversity covary spatially, we assessed the correlation between β‐GD and β‐SD in each ocean basin (Figure 3c). β species‐genetic diversity correlation was positive in the Western Indian Ocean using β_jac_‐SD (Figure S2b; Table 2; MLPE *p*‐value < 0.01) and marginally significant using β_jtu_‐SD (Figure 3c; Table 2; MLPE *p*‐value = 0.05), but never significant in the Caribbean Sea (Table 2; MLPE *p*‐value > 0.05).

## 4 Discussion

The spatial structure of habitats is recognized as a major determinant of connectivity between populations or communities by influencing how individuals move across space (Baguette et al. 2013; Cowman and Bellwood 2013; Riginos and Liggins 2013; Dalongeville et al. 2018). However, the influence of landscape or seascape connectivity on both intra‐ and interspecific diversity has rarely been examined in an integrated framework. Our study addressed this gap by evaluating the co‐variation of species and genetic β‐diversity in tropical fishes and comparing them in two distinct geographical regions: the Caribbean Sea and the Western Indian Ocean. Overall, we observed IBD in both genetic and species β‐diversity in the Western Indian Ocean, whereas the Caribbean Sea exhibits IBD only in species β‐diversity. These patterns confirm an overall positive correlation between species and genetic β‐diversity in the Western Indian Ocean (Vilcot et al. 2023). In contrast, the higher connectivity in the Caribbean Sea led to an absence of covariation between genetic and species β‐diversities. Together, our findings highlight the critical role of seascape structural connectivity in shaping functional connectivity from intra‐ to interspecific levels.

Positive β‐SGDCs have been reported in several natural systems (see Kahilainen et al. 2014), often related to connectivity (Vilcot et al. 2023), historical processes (Robuchon et al. 2019), or even to direct interaction between species and genetic diversity (Fourtune et al. 2016). Our results align with the findings of Baselga et al. (2013), who revealed consistent distance‐decay patterns from genetic to species when dispersal constrains diversity distribution. The Caribbean Sea, with its higher proportion of habitable area and greater number of habitat patches, demonstrates a much lower mean distance between patches compared to the Western Indian Ocean (Keggin 2023). This configuration likely facilitates higher dispersal among reef fish populations (Buonomo et al. 2017). Although we considered isolation based on geographical distance as a first approximation, the ocean dynamics can be particularly strong in some regions such as the Caribbean Sea. Incorporating isolation by temporal distance derived from ocean circulation models simulating particle movement, and extending the sampling to more ecoregions may reveal different results in the Caribbean Sea (White et al. 2010). A thorough assessment of larval connectivity that accounts for ocean currents could further help disentangle the relative contribution of currents versus geographic distance alone in shaping both genetic differentiation and community dissimilarities (e.g., Bernal‐Durán et al. 2024; Burt et al. 2024). Such approaches can also provide insights into the directionality of connectivity among communities (Riginos et al. 2016).

Our study leverages extensive multispecies genetic sampling. However, the combination of this genetic dataset with occurrence data that were not generated to address this specific research question may introduce heterogeneity in the spatial resolution of the detected patterns. Nevertheless, combining these complementary datasets provided a broad coverage of environmental heterogeneity. As the detection of genotype–environment relationships strongly depends on the spatial and temporal matching between environmental predictors, sampling design, and the biological scale at which adaptive processes occur (Dauphin et al. 2023), our results should be interpreted as reflecting broad‐scale trends rather than local‐scale processes. Additionally, the limited number of sites sampled in each ocean basin due to a trade‐off between sampling many sites or many species using traditional genetic data, constrained our ability to estimate spatial patterns at a high resolution. Leveraging standardized sampling strategies such as environmental DNA (e.g., Adams et al. 2019; Vilcot et al. 2026) would enable finer‐scale genetic datasets, facilitating comparisons of species‐specific and family‐specific diversity patterns.

In this study, we compared species from the same five families across the two ocean basins, ensuring broadly comparable dispersal potential and life‐history traits. Of the five families studied, three (Acanthuridae, Labridae, Pomacentridae) are among the six most dominant reef families according to the Coral Fish Diversity Index (Allen and Werner 2002), and thus play a key role in structuring reef fish communities. This focus on dominant families ensures that our findings reflect patterns relevant to the broader ecological dynamics of tropical reef ecosystems. Yet, tropical reef fishes exhibit a large disparity of life histories and ecological strategies, which significantly influence their dispersal (Cowman and Bellwood 2013; Bellwood et al. 2017). Dispersal, occurring during the pelagic larval stage for the majority of fishes (Leis 1991; Green et al. 2015), plays a pivotal role in population connectivity and gene flow among geographically separated populations in interaction with the seascape (Morrison et al. 2011). Key biological traits, such as PLD (Selkoe and Toonen 2011), swimming ability (Hogan et al. 2007), reproductive behaviors and parental investment (Riginos et al. 2014), or even the timing of the spawning (Torquato and Møller 2022) vary widely among species and critically impact the extent and success of dispersal and resulting diversity patterns. In the Western Indian Ocean, species with shorter PLD have been shown to exhibit stronger positive β‐SGDCs (Vilcot et al. 2023). However, when reef systems are sufficiently connected so that most sites are within the dispersal range of all species regardless of their dispersal capacities, the influence of PLD on diversity patterns is expected to weaken or disappear (Mora et al. 2012). This scenario is likely in the Caribbean Sea, where reef distances are generally smaller and connectivity higher. We hypothesize that species with lower dispersal ability may be more sensitive to seascape structure and show the greatest differences in β‐diversity between ocean basins. Further exploration of the interactions between species dispersal ability and habitat connectivity could help unravel the variability in IBD patterns across lineages, especially by estimating lineage‐specific dispersal abilities, or even dispersal kernel estimates rather than mean trait values (D'Aloia et al. 2015). In particular, combining biophysical models with β‐diversity estimates would help unravel the interplay between seascape structure and species dispersal abilities (Jahnke and Jonsson 2022), offering a more comprehensive and mechanistic picture of connectivity patterns from genes to communities.

In conclusion, our study based on a multispecies genetic sampling across two biogeographic regions highlights that intra‐ and interspecific biodiversity patterns and the covariation between them can be highly variable. We emphasize the major role of seascape connectivity on both population and community assembly in tropical reef fishes, suggesting intricate relationships between micro‐ and macroevolutionary biodiversity patterns.

## Author Contributions

**Maurine Vilcot:** conceptualization (equal), data curation (equal), formal analysis (lead), investigation (equal), visualization (lead), writing – original draft (lead), writing – review and editing (equal). **Thomas Keggin:** conceptualization (equal), data curation (equal), investigation (equal), writing – review and editing (equal). **Camille Albouy:** conceptualization (equal), funding acquisition (equal), investigation (equal), project administration (equal), resources (equal), writing – review and editing (equal). **Giulia F. A. Donati:** data curation (equal), investigation (equal), writing – review and editing (equal). **Andrés Acosta‐Chaparro:** investigation (equal), writing – review and editing (equal). **Giomar H. Borrero‐Pérez:** investigation (equal). **Marie‐Charlotte Cheutin:** investigation (equal), writing – review and editing (equal). **Juan David González Corredor:** investigation (equal), writing – review and editing (equal). **Régis Hocdé:** investigation (equal), writing – review and editing (equal). **Jean‐Baptiste Juhel:** investigation (equal), writing – review and editing (equal). **Virginie Marques:** investigation (equal), writing – review and editing (equal). **David Mouillot:** conceptualization (equal), funding acquisition (equal), writing – review and editing (equal). **Maria Mutis‐Martinezguerra:** investigation (equal), writing – review and editing (equal). **Andrea Polanco F:** funding acquisition (equal), investigation (equal), writing – review and editing (equal). **Mark J. A. Vermeij:** investigation (equal), writing – review and editing (equal). **Loïc Pellissier:** conceptualization (equal), funding acquisition (equal), investigation (equal), project administration (equal), resources (equal), writing – review and editing (equal). **Stéphanie Manel:** conceptualization (equal), funding acquisition (equal), project administration (equal), resources (equal), supervision (equal), writing – review and editing (equal).

## Funding

This work was funded by the Swiss National Science Foundation (SNF) and French National Research Agency (ANR) project “REEFISH” (no. 310030E‐164294). This work has also been funded by the PRCI SNF‐ANR project “SHIFTeDNA” through generic call (ANR no. 21‐CE02‐0032, SNF no. 205556).

## Ethics Statement

All the genetic data produced and analyzed in this paper are repurposed from Donati et al. (2021) and Keggin et al. (2023), and have no commercial value. Sampling was performed in accordance with local regulations and with local collaborators. Research permits are the following: Maldives ((OTHR) 30‐D/INDIV/2016/538); Mayotte (06/UTM/2016); Seychelles (A0157); Tanzania (2017–242‐NA‐2017‐87); Curaçao under sampling permit 2012/48,584 provided to CARMABI (Piscaderabaai 00000, Willemstad, Curaçao; a registered CITES Institution under Code AN001) by the Curaçaon Government; Martinique, decision number 122 of the director of the sea of Martinique. Colombian samples were collected in collaboration with INVEMAR, through which all samples were collected and exported as stated in the following: “According to Paragraph 1, Article 2.2.2.2.8.1.2. Section 1 (Permits), Chapter 8 (Scientific Research), of Decree 1076 of 2015.” The Ministry of Environment and Sustainable Development, its attached entities, National Natural Parks of Colombia, the Regional Autonomous Corporations and/or of Sustainable Development and the Large Urban Centers shall not require the Permit to Collect specimens referred to in this decree (…); “therefore, INVEMAR being an entity attached to the Ministry of Environment and Sustainable Development (see Article 1.2.2.1., Title 2, of Decree 1076 of 2015), does not require permission to collect specimens of wild species. In this sense, the material to be exported comes from projects developed by INVEMAR, which supports the legal acquisition of the specimens to be exported.”

## Conflicts of Interest

The authors declare no conflicts of interest.

## Supporting information


**Figure S1:** Number of samples sequenced for all the 42 species collected in the Caribbean Sea and the Western Indian Ocean.
**Figure S2:** MLPE linear mixed model of species isolation by distance (a) and β species‐genetic diversity correlation (b) using total Jaccard dissimilarity as species β‐diversity.
**Table S1:** Genotyped species excluded from the analysis due to insufficient sampling or lack of oceanic comparison.

## Data Availability

The scripts and data used for this article are available on Zenodo at https://doi.org/10.5281/zenodo.16982328.

## References

[ece373760-bib-0001] Adams, C. I. M. , M. Knapp , N. J. Gemmell , et al. 2019. “Beyond Biodiversity: Can Environmental DNA (eDNA) Cut It as a Population Genetics Tool?” Genes 10: 192. 30832286 10.3390/genes10030192 PMC6470983

[ece373760-bib-0002] Albouy, C. , P. Archambault , W. Appeltans , et al. 2019. “The Marine Fish Food Web is Globally Connected.” Nature Ecology & Evolution 3: 1153–1161. 31358950 10.1038/s41559-019-0950-y

[ece373760-bib-0003] Allen, G. R. , and T. B. Werner . 2002. “Coral Reef Fish Assessment in the ‘Coral Triangle’ of Southeastern Asia.” Environmental Biology of Fishes 65: 209–214.

[ece373760-bib-0004] Baguette, M. , S. Blanchet , D. Legrand , V. M. Stevens , and C. Turlure . 2013. “Individual Dispersal, Landscape Connectivity and Ecological Networks.” Biological Reviews 88: 310–326. 23176626 10.1111/brv.12000

[ece373760-bib-0005] Baselga, A. 2010. “Partitioning the Turnover and Nestedness Components of Beta Diversity: Partitioning Beta Diversity.” Global Ecology and Biogeography 19: 134–143.

[ece373760-bib-0006] Baselga, A. 2012. “The Relationship Between Species Replacement, Dissimilarity Derived From Nestedness, and Nestedness.” Global Ecology and Biogeography 21: 1223–1232.

[ece373760-bib-0007] Baselga, A. , T. Fujisawa , A. Crampton‐Platt , et al. 2013. “Whole‐Community DNA Barcoding Reveals a Spatio‐Temporal Continuum of Biodiversity at Species and Genetic Levels.” Nature Communications 4: 1892. 10.1038/ncomms2881 23695686

[ece373760-bib-0008] Baselga, A. , C. Gómez‐Rodríguez , and A. P. Vogler . 2015. “Multi‐Hierarchical Macroecology At Species and Genetic Levels to Discern Neutral and Non‐Neutral Processes: Multi‐Hierarchical Macroecology.” Global Ecology and Biogeography 24: 873–882.

[ece373760-bib-0009] Baselga, A. , and C. D. L. Orme . 2012. “ betapart: An R Package for the Study of Beta Diversity: *Betapart Package* .” Methods in Ecology and Evolution 3: 808–812.

[ece373760-bib-0010] Bellwood, D. R. , C. H. R. Goatley , and O. Bellwood . 2017. “The Evolution of Fishes and Corals on Reefs: Form, Function and Interdependence.” Biological Reviews of the Cambridge Philosophical Society 92: 878–901. 26970292 10.1111/brv.12259

[ece373760-bib-0011] Benestan, L. , K. Fietz , N. Loiseau , et al. 2021. “Restricted Dispersal in a Sea of Gene Flow.” Proceedings of the Royal Society B: Biological Sciences 288: 20210458. 10.1098/rspb.2021.0458 PMC8131118 34004134

[ece373760-bib-0012] Bernal‐Durán, V. , D. Donoso , A. Piñones , et al. 2024. “Combining Population Genomics and Biophysical Modelling to Assess Connectivity Patterns in an Antarctic Fish.” Molecular Ecology 33: e17360. 38656687 10.1111/mec.17360

[ece373760-bib-0013] Boulanger, E. , A. Dalongeville , M. Andrello , D. Mouillot , and S. Manel . 2020. “Spatial Graphs Highlight How Multi‐Generational Dispersal Shapes Landscape Genetic Patterns.” Ecography 43: 1167–1179.

[ece373760-bib-0014] Buonomo, R. , J. Assis , F. Fernandes , A. H. Engelen , L. Airoldi , and E. A. Serrão . 2017. “Habitat Continuity and Stepping‐Stone Oceanographic Distances Explain Population Genetic Connectivity of the Brown Alga Cystoseira Amentacea.” Molecular Ecology 26: 766–780. 27997043 10.1111/mec.13960

[ece373760-bib-0015] Burt, A. J. , N. Vogt‐Vincent , H. Johnson , et al. 2024. “Integration of Population Genetics With Oceanographic Models Reveals Strong Connectivity Among Coral Reefs Across Seychelles.” Scientific Reports 14: 4936. 38472289 10.1038/s41598-024-55459-x PMC10933301

[ece373760-bib-0016] Chang, J. , D. L. Rabosky , S. A. Smith , and M. E. Alfaro . 2019. “An r Package and Online Resource for Macroevolutionary Studies Using the Ray‐Finned Fish Tree of Life.” Methods in Ecology and Evolution 10: 1118–1124.

[ece373760-bib-0017] Clarke, R. T. , P. Rothery , and A. F. Raybould . 2002. “Confidence Limits for Regression Relationships Between Distance Matrices: Estimating Gene Flow With Distance.” Journal of Agricultural, Biological, and Environmental Statistics 7: 361–372.

[ece373760-bib-0018] Cowman, P. F. , and D. R. Bellwood . 2013. “The Historical Biogeography of Coral Reef Fishes: Global Patterns of Origination and Dispersal.” Journal of Biogeography 40: 209–224.

[ece373760-bib-0019] D'Aloia, C. C. , S. M. Bogdanowicz , R. K. Francis , J. E. Majoris , R. G. Harrison , and P. M. Buston . 2015. “Patterns, Causes, and Consequences of Marine Larval Dispersal.” Proceedings of the National Academy of Sciences of the United States of America 112: 13940–13945. 26508628 10.1073/pnas.1513754112 PMC4653176

[ece373760-bib-0020] Dalongeville, A. , M. Andrello , D. Mouillot , et al. 2018. “Geographic Isolation and Larval Dispersal Shape Seascape Genetic Patterns Differently According to Spatial Scale.” Evolutionary Applications 11: 1437–1447. 30151051 10.1111/eva.12638 PMC6099820

[ece373760-bib-0021] Dauphin, B. , C. Rellstab , R. O. Wüest , et al. 2023. “Re‐Thinking the Environment in Landscape Genomics.” Trends in Ecology & Evolution 38: 261–274. 36402651 10.1016/j.tree.2022.10.010

[ece373760-bib-0022] Donati, G. F. A. , C. Albouy , T. Claverie , et al. 2025. “Continuity in Morphological Disparity in Tropical Reef Fishes Across Evolutionary Scales.” Communications Biology 8: 1–10. 39966681 10.1038/s42003-025-07634-7 PMC11836192

[ece373760-bib-0023] Donati, G. F. A. , N. Zemp , S. Manel , et al. 2021. “Species Ecology Explains the Spatial Components of Genetic Diversity in Tropical Reef Fishes.” Proceedings of the Royal Society B: Biological Sciences 288: 20211574. 10.1098/rspb.2021.1574 PMC8479362 34583586

[ece373760-bib-0024] Fourtune, L. , I. Paz‐Vinas , G. Loot , J. G. Prunier , and S. Blanchet . 2016. “Lessons From the Fish: A Multi‐Species Analysis Reveals Common Processes Underlying Similar Species‐Genetic Diversity Correlations.” Freshwater Biology 61: 1830–1845.

[ece373760-bib-0025] Galindo, H. M. , D. B. Olson , and S. R. Palumbi . 2006. “Seascape Genetics: A Coupled Oceanographic‐Genetic Model Predicts Population Structure of Caribbean Corals.” Current Biology 16: 1622–1626. 16920623 10.1016/j.cub.2006.06.052

[ece373760-bib-0026] Green, A. L. , A. P. Maypa , G. R. Almany , et al. 2015. “Larval Dispersal and Movement Patterns of Coral Reef Fishes, and Implications for Marine Reserve Network Design.” Biological Reviews 90: 1215–1247. 25423947 10.1111/brv.12155

[ece373760-bib-0027] Hansen, M. M. , and J. Hemmer‐Hansen . 2007. “Landscape Genetics Goes to Sea.” Journal of Biology 6: 6. 18021427 10.1186/jbiol59 PMC2373898

[ece373760-bib-0028] Hedrick, P. W. 2005. “A Standardized Genetic Differentiation Measure.” Evolution 59: 1633–1638. 16329237

[ece373760-bib-0029] Hellberg, M. E. 2009. “Gene Flow and Isolation Among Populations of Marine Animals.” Annual Review of Ecology, Evolution, and Systematics 40: 291–310.

[ece373760-bib-0030] Hogan, J. D. , R. Fisher , and C. Nolan . 2007. “Critical Swimming Speed of Settlement‐Stage Coral Reef Fishes From the Caribbean: A Methodological and Geographical Comparison.” Bulletin of Marine Science 80: 219–231.

[ece373760-bib-0031] Huyghe, F. , and M. Kochzius . 2018. “Sea Surface Currents and Geographic Isolation Shape the Genetic Population Structure of a Coral Reef Fish in the Indian Ocean.” PLoS One 13: e0193825. 29522547 10.1371/journal.pone.0193825 PMC5844546

[ece373760-bib-0032] Jahnke, M. , and P. R. Jonsson . 2022. “Biophysical Models of Dispersal Contribute to Seascape Genetic Analyses.” Philosophical Transactions of the Royal Society, B: Biological Sciences 377: 20210024. 10.1098/rstb.2021.0024 PMC8784932 35067094

[ece373760-bib-0033] Kahilainen, A. , M. Puurtinen , and J. S. Kotiaho . 2014. “Conservation Implications of Species–Genetic Diversity Correlations.” Global Ecology and Conservation 2: 315–323.

[ece373760-bib-0034] Keggin, T. 2023. “The Emergence of Biological Diversity Through Organisational Scale.” PhD Thesis, ETH Zurich, Switzerland. 10.3929/ethz-b-000620696.

[ece373760-bib-0035] Keggin, T. , C. Waldock , A. Skeels , et al. 2023. “Diversity Across Organisational Scale Emerges Through Dispersal Ability and Speciation Dynamics in Tropical Fish.” BMC Biology 21: 282. 38053182 10.1186/s12915-023-01771-3 PMC10696697

[ece373760-bib-0036] Kimura, M. , and G. H. Weiss . 1964. “The Stepping Stone Model of Population Structure and the Decrease of Genetic Correlation With Distance.” Genetics 49: 561–576. 17248204 10.1093/genetics/49.4.561 PMC1210594

[ece373760-bib-0037] Kokko, H. , and A. López‐Sepulcre . 2006. “From Individual Dispersal to Species Ranges: Perspectives for a Changing World.” Science 313: 789–791. 16902127 10.1126/science.1128566

[ece373760-bib-0038] Kulbicki, M. , V. Parravicini , D. R. Bellwood , et al. 2013. “Global Biogeography of Reef Fishes: A Hierarchical Quantitative Delineation of Regions.” PLoS One 8: e81847. 24386083 10.1371/journal.pone.0081847 PMC3875412

[ece373760-bib-0039] Lamy, T. , F. Laroche , P. David , F. Massol , and P. Jarne . 2017. “The Contribution of Species–Genetic Diversity Correlations to the Understanding of Community Assembly Rules.” Oikos 126: 759–771.

[ece373760-bib-0040] Leis, J. M. 1991. “The Pelagic Stage of Reef Fishes: The Larval Biology of Coral Reef Fishes.” In The Ecology of Fishes on Coral Reefs, edited by P. F. Sale , 183–230. Academic Press.

[ece373760-bib-0041] Liggins, L. , E. A. Treml , H. P. Possingham , and C. Riginos . 2016. “Seascape Features, Rather Than Dispersal Traits, Predict Spatial Genetic Patterns in Co‐Distributed Reef Fishes.” Journal of Biogeography 43: 256–267.

[ece373760-bib-0042] Luiz, O. J. , A. P. Allen , D. R. Robertson , et al. 2013. “Adult and Larval Traits as Determinants of Geographic Range Size Among Tropical Reef Fishes.” Proceedings of the National Academy of Sciences of the United States of America 110: 16498–16502. 24065830 10.1073/pnas.1304074110 PMC3799316

[ece373760-bib-0043] Manel, S. , and R. Holderegger . 2013. “Ten Years of Landscape Genetics.” Trends in Ecology & Evolution 28: 614–621. 23769416 10.1016/j.tree.2013.05.012

[ece373760-bib-0044] McLean, M. , R. D. Stuart‐Smith , S. Villéger , et al. 2021. “Trait Similarity in Reef Fish Faunas Across the World's Oceans.” Proceedings of the National Academy of Sciences of the United States of America 118: e2012318118. 33723036 10.1073/pnas.2012318118 PMC7999973

[ece373760-bib-0045] Meirmans, P. G. , and P. W. Hedrick . 2011. “Assessing Population Structure: FST and Related Measures.” Molecular Ecology Resources 11: 5–18. 21429096 10.1111/j.1755-0998.2010.02927.x

[ece373760-bib-0046] Mora, C. , E. A. Treml , J. Roberts , K. Crosby , D. Roy , and D. P. Tittensor . 2012. “High Connectivity Among Habitats Precludes the Relationship Between Dispersal and Range Size in Tropical Reef Fishes.” Ecography 35: 89–96.

[ece373760-bib-0047] Morrison, R. A. , S. A. Sandin , R. A. Morrison , and S. A. Sandin . 2011. “Biogeography and Population Connectivity of Coral Reef Fishes.” In Changing Diversity in Changing Environment. IntechOpen.

[ece373760-bib-0048] Nei, M. 1987. Molecular Evolutionary Genetics. Columbia University Press.

[ece373760-bib-0049] Nekola, J. C. , and P. S. White . 1999. “The Distance Decay of Similarity in Biogeography and Ecology.” Journal of Biogeography 26: 867–878.

[ece373760-bib-0050] Olds, A. D. , R. M. Connolly , K. A. Pitt , et al. 2016. “Quantifying the Conservation Value of Seascape Connectivity: A Global Synthesis.” Global Ecology and Biogeography 25: 3–15.

[ece373760-bib-0051] Pante, E. , and B. Simon‐Bouhet . 2013. “Marmap: A Package for Importing, Plotting and Analyzing Bathymetric and Topographic Data in R.” PLoS One 8: e73051. 24019892 10.1371/journal.pone.0073051 PMC3760912

[ece373760-bib-0052] Parravicini, V. , M. G. Bender , S. Villéger , et al. 2021. “Coral Reef Fishes Reveal Strong Divergence in the Prevalence of Traits Along the Global Diversity Gradient.” Proceedings of the Royal Society B: Biological Sciences 288: 20211712. 10.1098/rspb.2021.1712 PMC8527194 34666520

[ece373760-bib-0053] Parravicini, V. , M. Kulbicki , D. R. Bellwood , et al. 2013. “Global Patterns and Predictors of Tropical Reef Fish Species Richness.” Ecography 36: 1254–1262.

[ece373760-bib-0054] Peterson, B. K. , J. N. Weber , E. H. Kay , H. S. Fisher , and H. E. Hoekstra . 2012. “Double Digest RADseq: An Inexpensive Method for De Novo SNP Discovery and Genotyping in Model and Non‐Model Species.” PLoS One 7: e37135. 22675423 10.1371/journal.pone.0037135 PMC3365034

[ece373760-bib-0055] R Core Team . 2022. R: A Language and Environment for Statistical Computing. R Foundation for Statistical Computing.

[ece373760-bib-0056] Rabosky, D. L. , J. Chang , P. O. Title , et al. 2018. “An Inverse Latitudinal Gradient in Speciation Rate for Marine Fishes.” Nature 559: 392–395. 29973726 10.1038/s41586-018-0273-1

[ece373760-bib-0057] Riginos, C. , Y. M. Buckley , S. P. Blomberg , and E. A. Treml . 2014. “Dispersal Capacity Predicts Both Population Genetic Structure and Species Richness in Reef Fishes.” American Naturalist 184: 52–64. 10.1086/676505 24921600

[ece373760-bib-0058] Riginos, C. , E. D. Crandall , L. Liggins , P. Bongaerts , and E. A. Treml . 2016. “Navigating the Currents of Seascape Genomics: How Spatial Analyses Can Augment Population Genomic Studies.” Current Zoology 62: 581–601. 29491947 10.1093/cz/zow067 PMC5804261

[ece373760-bib-0059] Riginos, C. , and L. Liggins . 2013. “Seascape Genetics: Populations, Individuals, and Genes Marooned and Adrift.” Geography Compass 7: 197–216.

[ece373760-bib-0060] Robuchon, M. , B. Leroy , C. Jézéquel , and B. Hugueny . 2019. “Correlations Between Broad‐Scale Taxonomic and Genetic Differentiations Suggest a Dominant Imprint of Historical Processes on Beta Diversities.” Journal of Biogeography 46: 1083–1095.

[ece373760-bib-0061] Selkoe, K. A. , C. C. D'Aloia , E. D. Crandall , et al. 2016. “A Decade of Seascape Genetics: Contributions to Basic and Applied Marine Connectivity.” Marine Ecology Progress Series 554: 1–19.

[ece373760-bib-0062] Selkoe, K. A. , and R. J. Toonen . 2011. “Marine Connectivity: A New Look at Pelagic Larval Duration and Genetic Metrics of Dispersal.” Marine Ecology Progress Series 436: 291–305.

[ece373760-bib-0063] Spalding, M. D. , H. E. Fox , G. R. Allen , et al. 2007. “Marine Ecoregions of the World: A Bioregionalization of Coastal and Shelf Areas.” Bioscience 57: 573–583.

[ece373760-bib-0064] Suárez, D. , P. Arribas , E. Jiménez‐García , and B. C. Emerson . 2022. “Dispersal Ability and Its Consequences for Population Genetic Differentiation and Diversification.” Proceedings of the Royal Society B: Biological Sciences 289: 20220489. 10.1098/rspb.2022.0489 PMC9115014 35582805

[ece373760-bib-0065] Torquato, F. , and P. R. Møller . 2022. “Physical–Biological Interactions Underlying the Connectivity Patterns of Coral‐Dependent Fishes Around the Arabian Peninsula.” Journal of Biogeography 49: 483–496.

[ece373760-bib-0066] UNEP‐WCMC, WorldFish Centre, WRI, & TNC . 2021. “Global Distribution of Warm‐Water Coral Reefs, Compiled From Multiple Sources Including the Millennium Coral Reef Mapping Project. Version 4.1.” UN Environment Programme World Conservation Monitoring Centre. 10.34892/t2wk-5t34.

[ece373760-bib-0067] Vellend, M. 2005. “Species Diversity and Genetic Diversity: Parallel Processes and Correlated Patterns.” American Naturalist 166: 199–215. 10.1086/431318 16032574

[ece373760-bib-0068] Vilcot, M. , C. Albouy , G. F. A. Donati , et al. 2023. “Spatial Genetic Differentiation Correlates With Species Assemblage Turnover Across Tropical Reef Fish Lineages.” Global Ecology and Biogeography 32: 535–547.

[ece373760-bib-0069] Vilcot, M. , M. Bruno , T. Keggin , et al. 2026. “A Continuum of Biodiversity: Revealing Marine Tropical Fish Diversity From Intra‐ to Interspecific Variation Through Environmental DNA.” Environmental DNA 8: e70298.

[ece373760-bib-0070] Vilcot, M. , N. Faure , K. R. Andrews , B. W. Bowen , F. Leprieur , and S. Manel . 2024. “Neutral Processes and Taxonomic Scale Drive Beta Species‐Genetic Diversity Correlations in a Submesophotic Tropical Reef Fish.” Molecular Ecology 33: e17423. 38825968 10.1111/mec.17423

[ece373760-bib-0071] White, C. , K. A. Selkoe , J. Watson , D. A. Siegel , D. C. Zacherl , and R. J. Toonen . 2010. “Ocean Currents Help Explain Population Genetic Structure.” Proceedings of the Royal Society B: Biological Sciences 277: 1685–1694. 10.1098/rspb.2009.2214 PMC2871860 20133354

[ece373760-bib-0072] Whittaker, R. H. 1960. “Vegetation of the Siskiyou Mountains, Oregon and California.” Ecological Monographs 30: 279–338.

[ece373760-bib-0073] Whittaker, R. H. 1972. “Evolution and Measurement of Species Diversity.” Taxon 21: 213–251.

[ece373760-bib-0074] Winter, D. J. 2012. “mmod: An R Library for the Calculation of Population Differentiation Statistics.” Molecular Ecology Resources 12: 1158–1160. 22883857 10.1111/j.1755-0998.2012.03174.x

[ece373760-bib-0075] Wright, S. 1943. “Isolation by Distance.” Genetics 28: 114–138. 17247074 10.1093/genetics/28.2.114 PMC1209196

