# Supplementary material for: Seascape Connectivity Shapes Genetic and Species β‐Diversity in Tropical Reef Fishes
Source: Ecol Evol. 2026 Jun 4;16(6):e73760. doi: 10.1002/ece3.73760 (PMC13238886; doi:10.1002/ece3.73760)
Supplement: Supplementary file 1 — Figure S1: Number of samples sequenced for all the 42 species collected in the Caribbean Sea and the Western Indian Ocean. Figure S2: MLPE linear mixed model of species isolation by distance (a) and β species‐genetic diversity correlation (b) using total Jaccard dissimilarity as species β‐diversity. Table S1: Genotyped species excluded from the analysis due to insufficient sampling or lack of oceanic comparison. [file ECE3-16-e73760-s001.docx]

Supporting Information for:

Seascape connectivity shapes genetic and species

β-diversity in tropical reef fishes

# APPENDIX


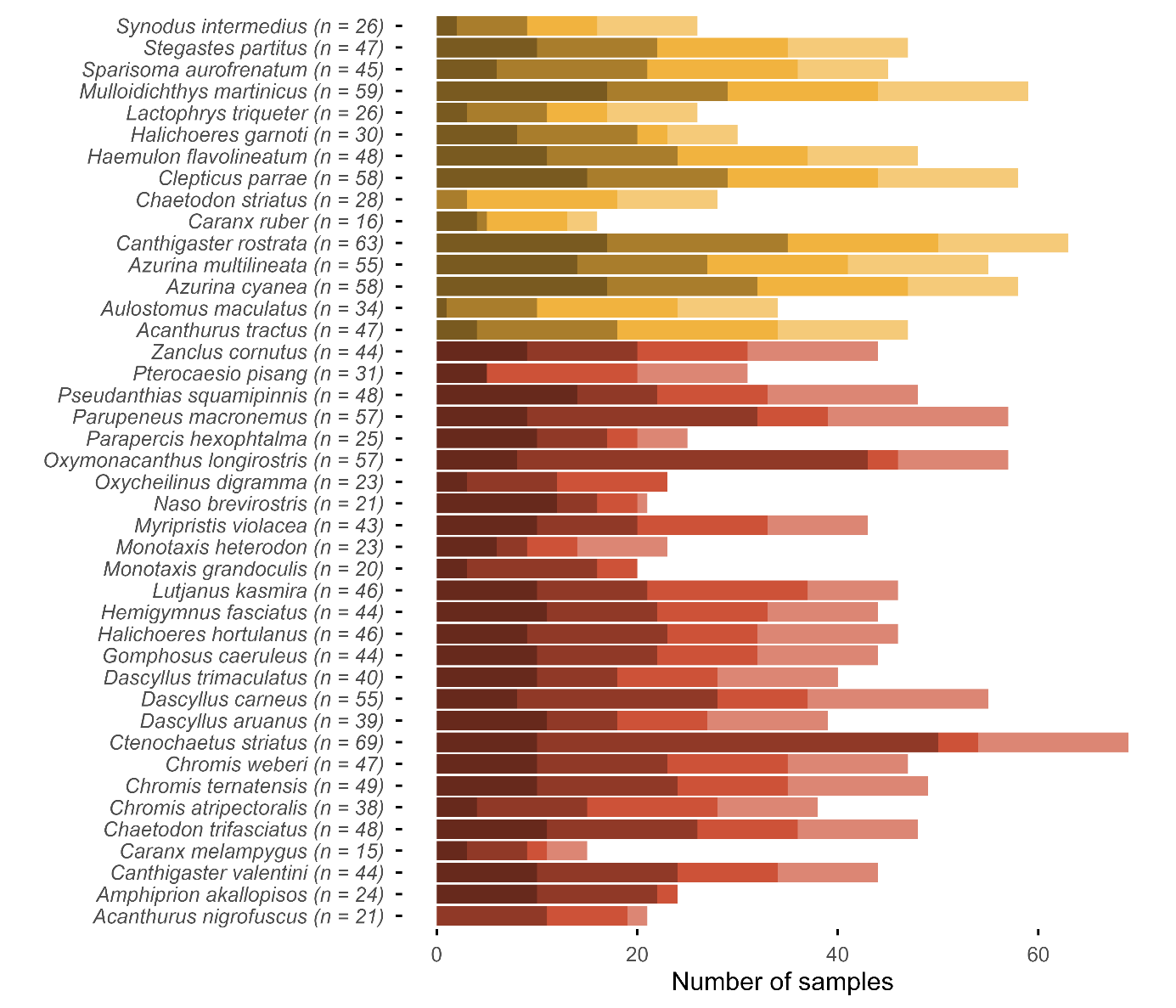


### **Figure A1.** Number of samples sequenced for all the 42 species collected in the Caribbean Sea (in orange, from darkest to lightest: Providencia, Santa Marta, Curaçao, Martinique) and the Western Indian Ocean (in red, from darkest to lightest: Mafia, Mayotte, Seychelles, Maldives)(Donati et al. 2021, Keggin 2023).


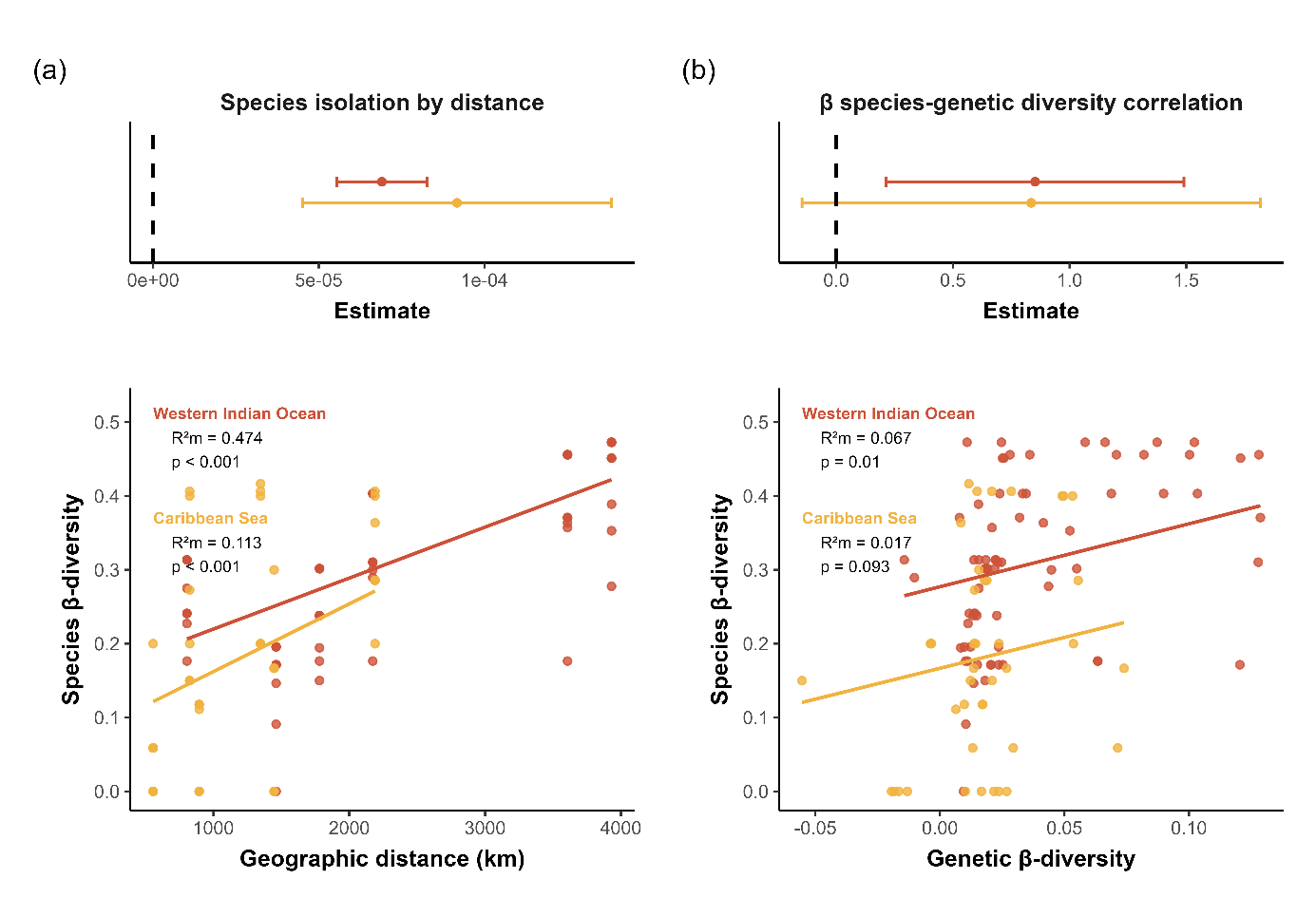


### **Figure A2.** MLPE linear mixed model of species isolation by distance (a) and β species-genetic diversity correlation (b) using total Jaccard dissimilarity as species β-diversity.

### **Table A1.** Genotyped species excluded from the analysis due to insufficient sampling or lack of oceanic comparison (i.e., no remaining species of the same family in the other ocean)(Keggin 2023).

| **Family** | **Ocean** | **Species** | **Reason** |
| --- | --- | --- | --- |
| Acanthuridae | Western Indian Ocean | *Acanthurus nigrofuscus* | Insufficient sampling |
| Acanthuridae | Western Indian Ocean | *Naso brevirostris* | Insufficient sampling |
| Aulostomidae | Caribbean Sea | *Aulostomus maculatus* | Insufficient sampling |
| Carangidae | Caribbean Sea | *Caranx ruber* | No oceanic comparison |
| Carangidae | Western Indian Ocean | *Caranx melampygus* | Insufficient sampling |
| Chaetodontidae | Caribbean Sea | *Chaetodon striatus* | Insufficient sampling |
| Chaetodontidae | Western Indian Ocean | *Chaetodon trifasciatus* | No oceanic comparison |
| Haemulidae | Caribbean Sea | *Haemulon flavolineatum* | No oceanic comparison |
| Holocentridae | Western Indian Ocean | *Myripristis violacea* | No oceanic comparison |
| Labridae | Western Indian Ocean | *Oxycheilinus digramma* | Insufficient sampling |
| Labridae | Western Indian Ocean | *Clepticus parrae* | Insufficient sampling |
| Lethrinidae | Western Indian Ocean | *Monotaxis grandoculis* | Insufficient sampling |
| Lethrinidae | Western Indian Ocean | *Monotaxis heterodon* | No oceanic comparison |
| Lutjanidae | Western Indian Ocean | *Lutjanus kasmira* | No oceanic comparison |
| Lutjanidae | Western Indian Ocean | *Pterocaesio pisang* | Insufficient sampling |
| Monacanthidae | Western Indian Ocean | *Oxymonacanthus longirostris* | No oceanic comparison |
| Ostraciidae | Caribbean Sea | *Lactophrys triqueter* | No oceanic comparison |
| Pinguipedidae | Western Indian Ocean | *Parapercis hexophtalma* | No oceanic comparison |
| Pomacentridae | Western Indian Ocean | *Amphiprion akallopisos* | Insufficient sampling |
| Scaridae | Caribbean Sea | *Sparisoma aurofrenatum* | No oceanic comparison |
| Serranidae | Western Indian Ocean | *Pseudanthias squamipinnis* | No oceanic comparison |
| Synodontidae | Caribbean Sea | *Synodus intermedius* | No oceanic comparison |
| Zanclidae | Western Indian Ocean | *Zanclus cornutus* | No oceanic comparison |
